# Multiple evanescent white dot syndrome following vaccination for COVID-19: A case report

**DOI:** 10.1097/MD.0000000000028582

**Published:** 2022-01-14

**Authors:** Sayako Inagawa, Masahiro Onda, Taishi Miyase, Shiho Murase, Hiroki Murase, Kiyofumi Mochizuki, Hirokazu Sakaguchi

**Affiliations:** a Department of Ophthalmology, Gifu University Graduate School of Medicine, Gifu, Japan; b Murase Eye Clinic, Gero, Japan.

**Keywords:** COVID-19, mRNA vaccine, multiple evanescent white dot syndrome

## Abstract

**Rationale:**

Multiple evanescent white dot syndrome (MEWDS) is an acute, usually unilateral, retinal disorder of unknown etiology that predominantly occurs in healthy young women. We report a case of bilateral asymmetric MEWDS that developed following the first vaccination for coronavirus-19 and worsened after a second vaccination.

**Patient concerns:**

A 30-year-old Japanese woman was examined in an eye clinic for blurred vision in her left eye for 1 week duration. Thirteen days before her examination, she had received her first BNT162b2 mRNA SARS-CoV-2 vaccination. Her best-corrected visual acuity was 20/20 in both eyes. Fundus examination revealed multiple yellowish-white spots in the perifoveal area of both eyes. Visibility of the spots gradually decreased during the following week. She was then vaccinated with a second dose, and 3 days later, her vision worsened in her left eye. She was then referred to our hospital because of worsened vision and the appearance of white spots on other parts of the retina. Ophthalmological examination revealed a best-corrected visual acuity of 30/20 both eyes.

**Diagnosis:**

The flare value in the anterior chamber was elevated in both the eyes. Fundus examination showed multiple white spots in the perifoveal area of both eyes, but they were more prominent in the left eye. Fundus fluorescein angiography revealed early hyperfluorescent spots located circumferentially around the fovea in both eyes. We concluded that the patient had MEWDS, which was most likely due to mRNA COVID-19 immunization.

**Interventions:**

The patient was treated with topical betamethasone sodium phosphate/fradiomycin sulfate 0.1% thrice daily for 2 months.

**Outcomes:**

Two months after treatment, her blurry vision resolved with the disappearance of the fundus lesions.

**Lesson:**

Clinicians should be aware of potential adverse ocular events following similar vaccinations.

## 1 Introduction

Multiple evanescent white dot syndrome (MEWDS) is an acute, usually unilateral, retinal disorder of unknown etiology that predominantly occurs in healthy young myopic women.^[1]^ Recently, Rabinovitch et al^[2]^ reported 2 patients who developed unilateral MEWDS after a second vaccination for coronavirus-19 (BNT162b2 mRNA). There have been other reports on the development of MEWDS after vaccination against rabies, human papilloma virus, hepatitis A, hepatitis B, meningococcal, yellow fever, and influenza.^[3–9]^

Here, we report our findings in a healthy Japanese woman who presented with bilateral asymmetric MEWDS that developed after her first BNT162b2 mRNA vaccination. After resolution of the blurred vision, she received a second vaccination, which led to the development of worse blurry vision.

## 2 Case report

**Figure 1:**
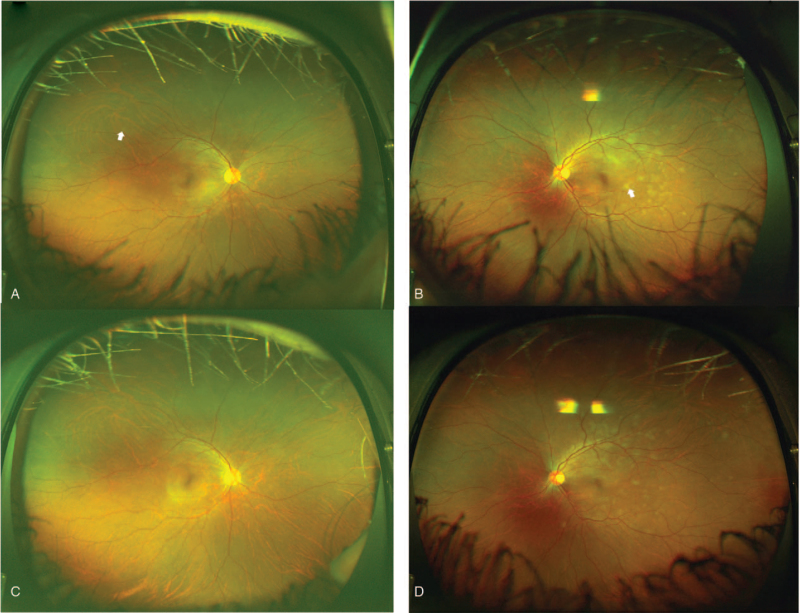
Fundus photographs of a 30-year-old woman who complained of blurred vision. Fundus examination of each eye showed multiple yellowish-white spots (white arrow) with more in the left (A) than the right (B) eye at the initial visit. One week later, the brightness of the yellowish-white spots decreased (C and D).

A 30-year-old healthy female nurse who worked in the general ward but not in the intensive care unit or pediatric unit visited an eye clinic complaining of blurred vision in her left eye that began 1 week earlier. Thirteen days before her examination, she had received her first BNT162b2 mRNA SARS-CoV-2 vaccination. She reported soreness at the injection site, but the pain disappeared within 24 hours. She had no other adverse reactions such as fatigue or fever. Her best-corrected visual acuity (BCVA) was 20/20 both eyes (OU). Anterior segment examination and visual field testing with a Humphrey Field Analyzer 30-2 grid were within normal limits in both eyes. Fundus examination showed multiple yellowish-white spots in the perifovea area, with more spots in the left eye than in the right eye (Fig. 1A and B). The patient was followed up with a topical corticosteroid (0.1% fluorometholone thrice daily for 21 days) with a diagnosis of presumed acute posterior multifocal placoid pigment epitheliopathy. Unfortunately, fundus fluorescein angiography was not performed. One week later, the visibility of yellowish-white spots decreased (Fig. 1C and D).

The following day, the patient received a second vaccination. She had fever (37.7°C), joint pain, and fatigue for 24 hours after the injection, but these symptoms were alleviated within 1 day by acetaminophen. Three days after the second injection, the patient presented to the clinic with a blurry vision in her left eye. White spots were seen as originally seen and were also detected in other parts of the retina of both eyes. She was referred to our hospital for further analysis and treatment.

**Figure 2:**
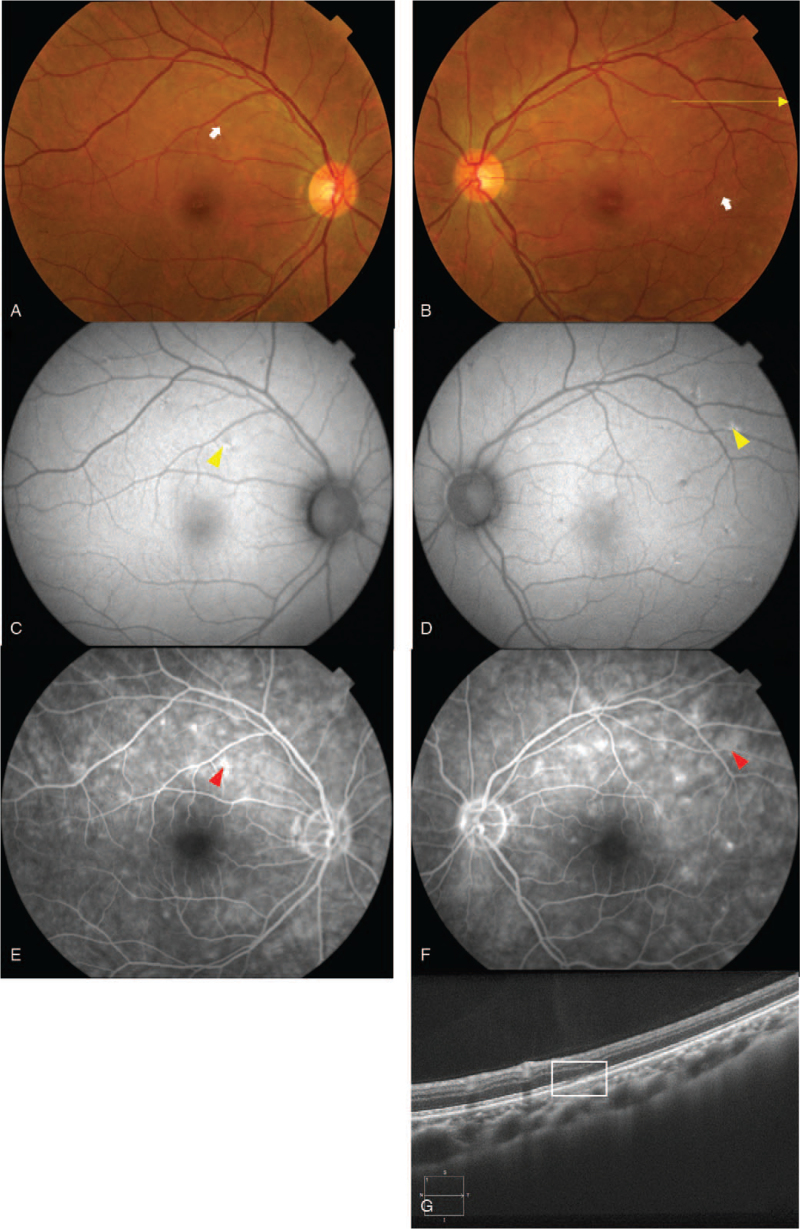
Fundus photographs, fundus autofluorescence, fundus fluorescein angiogram, and optical coherence tomographic (OCT) images recorded at our hospital after a second vaccination for COVID-19. The fundus examination showed multiple white spots (white arrow) in the perifovea area, superior, inferior and temporal to the fovea in both eyes. They are more prominent in the left eye (A and B). Fundus autofluorescence shows hyperfluorescent dots (yellow arrowhead) in the macular area (C and D). Fundus fluorescein angiography of each eye shows some early hyperfluorescent spots (red arrowhead) located circumferentially around the fovea (E and F). OCT image shows a disruption of the ellipsoid zone of the photoreceptors inferior to the fovea (white box) of the left eye (G). The horizontal yellow arrow in image B indicates the scanned area for the OCT shown in image G.

Ophthalmologic examination showed that her BCVA was 30/20 OU and intraocular pressure was 13 mm Hg OU. The pupils were equal, round, and reactive, without afferent pupillary defects. The extraocular eye movements were full. Slit-lamp examination revealed no anterior chamber inflammation in either eye, but the flare value in the anterior chamber was elevated to 18 photons/ms in the right eye and 16 photons/ms in the left eye (typical normal value, ≤5 photons/ms). Dilated fundus examination revealed a trace of vitreous cells in the right eye, 1+ vitreous cells in the left eye, and multiple white spots in the perifoveal area, superior, inferior, and temporal to the fovea in both eyes, but more prominently in the left eye (Fig. 2A and B). Fundus fundus autofluorescence of each eye showed hyperfluorescent dots in the macular area (Fig. 2C and D). Fundus fluorescein angiography showed early hyperfluorescent spots circumferentially around the fovea (Fig. 2E and F). Optical coherence tomography (OCT) showed disruption of the ellipsoid zone superior to the fovea (Fig. 2G). No indocyanine green angiography was performed. Visual field testing with the Humphrey Field Analyzer 30-2 grid showed no abnormalities, such as Marriott blind-spot enlargement in both eyes. Electroretinograms were normal. Systemic examinations, including routine blood tests and serologic tests for syphilis and hepatitis B and C, were all negative. She had not experienced any of the usual symptoms of COVID-19 and had never taken care of COVID-19 patients. Based on the ocular manifestations, she was diagnosed with MEWDS and continued treatment with topical corticosteroids (betamethasone sodium phosphate/fradiomycin sulfate 0.1% 3 times daily for 2 months).

At her 2-month follow-up visit, her symptom of blurry vision was resolved without any recurrences, the BCVA was still 30/20, the flare value was within normal limits, and the fundus lesions were absent. Changes in fundus autofluorescence and optical coherence tomography findings were still observed. Currently, no medication is being used.

The level of IgG (S-receptor-binding domain) determined by a chemiluminescent assay was 5663.8 AU/mL (positive threshold = 50 AU/mL) 14 days after the second vaccination.

## 3 Discussion

To the best of our knowledge, this is only the second report of a case of MEWDS that developed after a BNT162b2 mRNA vaccination and the first report of bilateral MEWDS following the first vaccination. The previous case involved a healthy 28-year-old woman with an onset 5 days after the second vaccination for COVID-19 and a 39-year-old man with an onset 30 days after the second vaccination. MEWDS findings were present only in the left eye and resolved without medication.^[2]^

Rabinovitch et al^[2]^ summarized similar reported cases of vaccination-associated MEWDS that developed in healthy young to middle-aged women with a median onset of 2 weeks after the vaccination (range, 1–30 days), mostly occurring in the left eye, the most common symptom was photophobia, the most common sign was a central or paracentral scotoma, and all had good visual outcomes with spontaneous resolution in 1 to 3 months. Our patient was a young woman who developed blurred vision only in her left eye 7 days after her first vaccination, and the blurred vision and number of white spots increased after the second vaccination. She was treated with topical corticosteroids because the flare value in the anterior chamber was elevated, and at the 2-month follow-up examination, the flare value was normal with the disappearance of retinal white spots.

The exact pathophysiology of MEWDS has not yet been conclusively determined. The most recent hypothesis is that it is due to an immune-mediated mechanism manifested in either the outer retina, choriocapillaris/inner choroid, or both, in genetically predisposed individuals.^[9]^ The possible pathophysiological mechanism for the mRNA vaccine to cause MEWDS has been divided into 2 steps. The first is an increase in the secretion of type-I interferons and the presence of extracellular RNA. The vaccine mRNA activates intracellular RNA-sensing molecules, which results in increased secretion of type-I interferon. The second step involves the enhancement of autoimmune manifestations and inflammation by an increase in type I interferon levels.^[10]^ The extracellular naked RNA increases the permeability of endothelial cells,^[10]^ especially the choriocapillaris and inner choroid.

Several inflammatory events other than MEWDS have been reported after COVID-19 vaccination. Rabinovitch et al^[2]^ described 19 patients with anterior uveitis (2 with bilateral inflammation) that developed after vaccination with BNT162b2 mRNA, with 8 cases occurring after the first vaccination and 11 cases after the second vaccination. In our case, bilateral inflammation of the anterior segment was observed, manifested by an increased flare in the anterior chamber. It was suggested that the immunizations may have contributed to uveitis similar to that of MEWDS.^[2]^ There are 3 other reports of posterior events after COVID-19 vaccination: a 43-year-old woman had a severe reactivation of Vogt-Koyanagi-Harada disease 6 weeks after the second dose of BNT162b2 mRNA SARS-CoV-2 vaccination,^[11]^ a 33-year-old man presented with central serous retinopathy in the right eye 3 days after the first injection of BNT162b2 mRNA vaccine^[12]^; and a 27-year-old healthy woman developed bilateral acute macular neuroretinopathy (AMN) 3 days after receiving the first AstraXeneca ChAdOx1-S (Vaxzevria: recombinant).^[13]^ In addition, there was 1 case of paracentral acute middle maculopathy, 2 cases of AMN, and 1 case of subretinal fluid after the first inoculation with the covid vaccine (Sinopharm: inactivated vaccine).^[14]^ There have also been 2 reports of anterior events: Descemet membrane endothelial keratoplasty allograft rejection and acute corneal graft rejection after BNT162b2 mRNA vaccination.^[15,16]^ It has been suggested that the allogeneic response may have been initiated by the host's antibody response following vaccination.^[15]^ Pichi et al^[14]^ reported scleritis and episcleritis in 4 cases at a mean of 5 days after the first dose of the COVID-19 vaccine (Sinopharm). Because similar ocular conditions, viz., paracentral acute middle maculopathy, AMN, graft rejection, and episcleritis, have been reported in patients with COVID-19,^[17,18]^ a polymerase chain reaction (PCR) test for SARS-CoV-2 may be needed to rule out COVID-19.

The most adverse systemic effects of the BNT162b2 mRNA vaccine generally appeared after the second dose.^[2]^ Similarly, in our case, systemic events of fever, joint pain, fatigue, and blurry vision in her left eye appeared after the second vaccination.

A limitation of this study is that PCR testing for SARS-CoV-2 was not performed. Because MEWDS is usually mild and self-limiting, it may be possible that more cases of MEDWS developed after COVID-19 vaccination, which were not noticed and thus not reported. Given the significant personal and public health benefits of vaccination for COVID-19 and the rare occurrence and low risk of ocular side effects, we do not suggest withholding vaccines in this population. However, clinicians should be aware of this possible association and encourage patients to pursue ophthalmic reviews to develop ocular adverse events.

## 4 Conclusion

In conclusion, MEWDS may be associated with vaccination with the mRNA COVID-19 vaccine. Close follow-up of young women with or without a history of uveitis after the administration of the COVID-19 vaccine is important to assess the visual signs and symptoms of MEWDS.

## Acknowledgments

We thank Professor Emeritus Duco Hamasaki of the Bascom Palmer Eye Institute for discussions and editing this manuscript.

## Author contributions

All authors have approved the paper. All authors attest that they met the current ICMJE criteria for authorship.

**Conceptualization:** Sayako Inagawa, Kiyofumi Mochizuki.

**Data curation:** Sayako Inagawa, Taishi Miyase, Shiho Murase, Hiroki Murase.

**Formal analysis:** Sayako Inagawa, Masahiro Onda, Taishi Miyase.

**Investigation:** Masahiro Onda, Shiho Murase, Hiroki Murase.

**Supervision:** Hirokazu Sakaguchi.

**Writing – original draft:** Sayako Inagawa.

**Writing – review & editing:** Kiyofumi Mochizuki, Hirokazu Sakaguchi.
